# Bis[1,3-bis(1-propyl-1*H*-benzimidazol-2-yl)-2-oxapropane]­cadmium(II) dipicrate dimethyl­formamide monosolvate

**DOI:** 10.1107/S1600536811038712

**Published:** 2011-09-30

**Authors:** Huilu Wu, Fan Kou, Fei Jia, Bin Liu, Jingkun Yuan

**Affiliations:** a Key Laboratory of Synthetic and Natural Functional Molecule Chemistry of the Ministry of Education, College of Chemistry & Materials Science, Northwest University, Xi’an 710069, People’s Republic of China; b School of Chemical and Biological Engineering, Lanzhou Jiaotong University, Lanzhou 730070, People’s Republic of China

## Abstract

In the title compound, [Cd(C_22_H_26_N_4_O)_2_](C_6_H_2_N_3_O_7_)_2_·C_3_H_7_NO, the Cd^II^ ion is coordinated by four N atoms and two O atoms from two tridentate 1,3-bis­(1-propyl-1*H*-benzimidazol-2-yl)-2-oxopropane ligands in a distorted octa­hedral coordination environment. There are significant differences in the chemically equivalent Cd—O bond lengths [2.618 (2) Å and 2.561 (2) Å].

## Related literature

For related structures, see: Addison *et al.* (1983 ▶); Wu, Kou *et al.* (2011 ▶); Wu, Liu *et al.* (2011 ▶). 
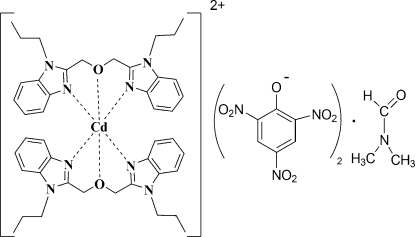


## Experimental

### 

#### Crystal data

[Cd(C_22_H_26_N_4_O)_2_](C_6_H_2_N_3_O_7_)_2_·C_3_H_7_NO
*M* *_r_* = 1366.64
Triclinic, 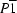

*a* = 14.211 (6) Å
*b* = 14.997 (7) Å
*c* = 16.190 (7) Å
α = 94.207 (4)°
β = 112.449 (4)°
γ = 97.986 (4)°
*V* = 3128 (2) Å^3^
*Z* = 2
Mo *K*α radiation
μ = 0.43 mm^−1^
*T* = 293 K
0.34 × 0.29 × 0.26 mm

#### Data collection

Bruker APEXII CCD diffractometer
Absorption correction: multi-scan (*SADABS*; Bruker, 2007 ▶) *T*_min_ = 0.867, *T*_max_ = 0.896
18954 measured reflections
10829 independent reflections
8918 reflections with *I* > 2σ(*I*)
*R*_int_ = 0.017

#### Refinement

*R*[*F* ^2^ > 2σ(*F* ^2^)] = 0.037
*wR*(*F* ^2^) = 0.106
*S* = 0.85
10829 reflections
835 parameters
3 restraints
H-atom parameters constrained
Δρ_max_ = 0.68 e Å^−3^
Δρ_min_ = −0.51 e Å^−3^

### 

Data collection: *APEX2* (Bruker, 2007 ▶); cell refinement: *SAINT* (Bruker, 2007 ▶); data reduction: *SAINT*; program(s) used to solve structure: *SHELXS97* (Sheldrick, 2008) ▶; program(s) used to refine structure: *SHELXL97* (Sheldrick, 2008) ▶; molecular graphics: *SHELXTL* (Sheldrick, 2008) ▶; software used to prepare material for publication: *SHELXTL*.

## Supplementary Material

Crystal structure: contains datablock(s) I. DOI: 10.1107/S1600536811038712/lh5332sup1.cif

Structure factors: contains datablock(s) I. DOI: 10.1107/S1600536811038712/lh5332Isup2.hkl

Additional supplementary materials:  crystallographic information; 3D view; checkCIF report

## Figures and Tables

**Fig. 1.**
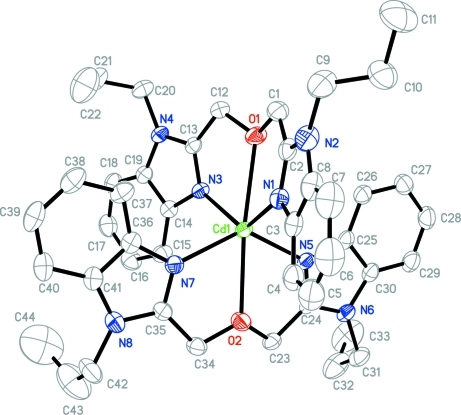
The molecular structure of the cation. Hydrogen atoms have been omitted for clarity and the displacement ellipsoids are shown at the 30% probability level.

## supplementary crystallographic information

### Comment

Interest in bis(2-benzimidazolyl)alkanes and their derivatives is widespread (Addison *et al.*, 1983). We have previously reported the crystal structure of some related complexes (Wu, Kou *et al.*, 2011; Wu, Liu *et al.*, 2011). The asymmetric unit of the title compound, consists of a discrete [Cd(1,3-bis(1-propyl-1*H*-benzimidazol-2-yl)-2-oxapropane)_2_] cation, two picrate anions and a dimethylformamide solvent molecule. The cation is shown in Fig. 1. The Cd^II^ ion is six-coordinate with a N_4_O_2_ ligand set. The ligand acts as a tridentate N-donor and O-donor. The coordination geometry of the Cd^II^ may be best described as distorted octahedral where four coordinated N atoms do not form an ideal equatorial plane. The axial sites are occupied by O1 and O2. The Cd—O bond distances indicate weak coordination. The crystal structure contains weak π···π  stacking interactions with centroid-to-centroid distances in the range 3.646 (3) - 3.795 (3)Å.

### Experimental

To a stirred solution of 1,3-bis(1-propyl-1*H*-benzimidazol-2-yl)-2-oxapropane (0.145 g, 0.4 mmol) in hot MeOH (5 ml),  Cd(C_6_H_2_N_3_O_7_)_2_ (0.114 g, 0.2 mmol) in MeOH (5 ml) was added. A yellow crystalline product formed rapidly. The precipitate was filtered off, washed with MeOH and absolute Et_2_O, and dried *in vacuo*. The dried precipitate was dissolved in acetonitrile to give a white solution which was allowed to evaporate at room temperature. The white crystals suitable for X-ray diffraction studies were obtained after three weeks. Yield, 0.213 g (78%).

### Refinement

All H atoms were found in difference electron maps and were subsequently refined in a riding-model approximation with C—H distances ranging from 0.95 to 0.97 Å and *U*_iso_(H) = 1.2 - 1.5 *U*_eq_ of the carrier atom,

### Crystal data

**Table:**

[Cd(C_22_H_26_N_4_O)_2_](C_6_H_2_N_3_O_7_)_2_·C_3_H_7_NO	*Z* = 2
*M**_r_* = 1366.64	*F*(000) = 1412
Triclinic, *P*1	*D*_x_ = 1.451 Mg m^−^^3^
Hall symbol: -P 1	Mo *K*α radiation, λ = 0.71073 Å
*a* = 14.211 (6) Å	Cell parameters from 8580 reflections
*b* = 14.997 (7) Å	θ = 2.3–25.9°
*c* = 16.190 (7) Å	µ = 0.43 mm^−^^1^
α = 94.207 (4)°	*T* = 293 K
β = 112.449 (4)°	Block, white
γ = 97.986 (4)°	0.34 × 0.29 × 0.26 mm
*V* = 3128 (2) Å^3^	

### Data collection

**Table:**

Bruker APEXII CCD diffractometer	10829 independent reflections
Radiation source: fine-focus sealed tube	8918 reflections with *I* > 2σ(*I*)
graphite	*R*_int_ = 0.017
ω scans	θ_max_ = 25.0°, θ_min_ = 2.1°
Absorption correction: multi-scan (*SADABS*; Bruker, 2007)	*h* = −16→12
*T*_min_ = 0.867, *T*_max_ = 0.896	*k* = −17→17
18954 measured reflections	*l* = −19→19

### Refinement

**Table:**

Refinement on *F*^2^	Primary atom site location: structure-invariant direct methods
Least-squares matrix: full	Secondary atom site location: difference Fourier map
*R*[*F*^2^ > 2σ(*F*^2^)] = 0.037	Hydrogen site location: inferred from neighbouring sites
*wR*(*F*^2^) = 0.106	H-atom parameters constrained
*S* = 0.85	*w* = 1/[σ^2^(*F*_o_^2^) + (0.057*P*)^2^ + 4.5262*P*] where *P* = (*F*_o_^2^ + 2*F*_c_^2^)/3
10829 reflections	(Δ/σ)_max_ = 0.002
835 parameters	Δρ_max_ = 0.68 e Å^−^^3^
3 restraints	Δρ_min_ = −0.51 e Å^−^^3^

### Special details

**Table:**

Geometry. All esds (except the esd in the dihedral angle between two l.s. planes) are estimated using the full covariance matrix. The cell esds are taken into account individually in the estimation of esds in distances, angles and torsion angles; correlations between esds in cell parameters are only used when they are defined by crystal symmetry. An approximate (isotropic) treatment of cell esds is used for estimating esds involving l.s. planes.
Refinement. Refinement of F^2^ against ALL reflections. The weighted R-factor wR and goodness of fit S are based on F^2^, conventional R-factors R are based on F, with F set to zero for negative F^2^. The threshold expression of F^2^ > 2sigma(F^2^) is used only for calculating R-factors(gt) etc. and is not relevant to the choice of reflections for refinement. R-factors based on F^2^ are statistically about twice as large as those based on F, and R- factors based on ALL data will be even larger.

### Fractional atomic coordinates and isotropic or equivalent isotropic displacement parameters (Å^2^)

**Table:**

	*x*	*y*	*z*	*U*_iso_*/*U*_eq_	
Cd1	0.710319 (17)	0.270821 (14)	0.087796 (14)	0.03937 (8)	
O1	0.6256 (2)	0.19157 (15)	0.18774 (14)	0.0565 (6)	
O2	0.77052 (18)	0.35207 (17)	−0.02188 (15)	0.0564 (6)	
N5	0.59304 (19)	0.34126 (16)	−0.00581 (16)	0.0410 (6)	
N4	0.62449 (19)	−0.02374 (16)	0.07041 (17)	0.0432 (6)	
N3	0.65589 (19)	0.11966 (16)	0.04931 (16)	0.0399 (6)	
N1	0.7491 (2)	0.35220 (18)	0.22203 (17)	0.0476 (6)	
N7	0.86953 (19)	0.25298 (17)	0.09737 (17)	0.0440 (6)	
N6	0.5179 (2)	0.41112 (17)	−0.12391 (18)	0.0478 (6)	
N8	0.9983 (2)	0.2479 (2)	0.05235 (18)	0.0522 (7)	
C24	0.6019 (2)	0.37620 (19)	−0.0757 (2)	0.0422 (7)	
C13	0.6321 (2)	0.06455 (19)	0.10070 (19)	0.0386 (6)	
C14	0.6656 (2)	0.0642 (2)	−0.01885 (19)	0.0418 (7)	
C23	0.6946 (2)	0.3787 (2)	−0.0974 (2)	0.0475 (7)	
H23A	0.6791	0.3371	−0.1516	0.057*	
H23B	0.7183	0.4395	−0.1067	0.057*	
C19	0.6460 (2)	−0.0258 (2)	−0.0062 (2)	0.0439 (7)	
C25	0.4979 (2)	0.35542 (19)	−0.0071 (2)	0.0431 (7)	
C35	0.9086 (2)	0.2777 (2)	0.0390 (2)	0.0441 (7)	
C12	0.6173 (3)	0.0960 (2)	0.1835 (2)	0.0454 (7)	
H12A	0.5497	0.0681	0.1798	0.054*	
H12B	0.6699	0.0803	0.2365	0.054*	
C40	1.0970 (3)	0.1515 (3)	0.1666 (3)	0.0693 (11)	
H40	1.1508	0.1486	0.1478	0.083*	
C30	0.4502 (2)	0.3986 (2)	−0.0814 (2)	0.0482 (8)	
C34	0.8581 (3)	0.3308 (2)	−0.0348 (2)	0.0515 (8)	
H34A	0.9051	0.3859	−0.0312	0.062*	
H34B	0.8376	0.2950	−0.0932	0.062*	
C36	0.9368 (2)	0.2022 (2)	0.1517 (2)	0.0477 (8)	
C9	0.7450 (4)	0.3742 (3)	0.4461 (3)	0.0899 (15)	
H9A	0.8111	0.3896	0.4973	0.108*	
H9B	0.7159	0.3115	0.4451	0.108*	
C18	0.6473 (3)	−0.0968 (2)	−0.0658 (2)	0.0553 (8)	
H18	0.6342	−0.1571	−0.0572	0.066*	
C3	0.8162 (3)	0.4348 (2)	0.2601 (2)	0.0538 (8)	
C15	0.6880 (3)	0.0866 (2)	−0.0918 (2)	0.0544 (8)	
H15	0.7015	0.1468	−0.1005	0.065*	
C41	1.0176 (3)	0.1987 (2)	0.1242 (2)	0.0519 (8)	
C26	0.4515 (3)	0.3358 (2)	0.0525 (2)	0.0522 (8)	
H26	0.4836	0.3076	0.1025	0.063*	
N2	0.7619 (3)	0.3831 (2)	0.36200 (19)	0.0661 (9)	
C2	0.7189 (3)	0.3245 (2)	0.2849 (2)	0.0523 (8)	
O7	0.1396 (3)	0.2479 (3)	0.4676 (2)	0.1135 (13)	
C20	0.6022 (3)	−0.1027 (2)	0.1122 (3)	0.0619 (9)	
H20A	0.5425	−0.1439	0.0679	0.074*	
H20B	0.5835	−0.0825	0.1612	0.074*	
O3	0.2767 (3)	0.4297 (2)	0.1884 (2)	0.0880 (9)	
C5	0.9307 (3)	0.5714 (3)	0.2791 (4)	0.0860 (14)	
H5	0.9666	0.6127	0.2566	0.103*	
C46	0.1506 (3)	0.4255 (2)	0.2484 (2)	0.0566 (9)	
C16	0.6898 (3)	0.0169 (3)	−0.1503 (2)	0.0611 (9)	
H16	0.7053	0.0300	−0.1995	0.073*	
C47	0.1043 (3)	0.3946 (2)	0.3020 (2)	0.0595 (9)	
H47	0.0434	0.4125	0.2997	0.071*	
N9	0.1061 (3)	0.4928 (3)	0.1904 (3)	0.0738 (9)	
C49	0.2395 (3)	0.3096 (2)	0.3641 (3)	0.0660 (10)	
H49	0.2701	0.2711	0.4050	0.079*	
C17	0.6686 (3)	−0.0735 (3)	−0.1375 (2)	0.0626 (10)	
H17	0.6691	−0.1194	−0.1792	0.075*	
C8	0.8242 (3)	0.4543 (3)	0.3482 (2)	0.0648 (10)	
C6	0.9400 (4)	0.5894 (4)	0.3674 (4)	0.0992 (17)	
H6	0.9829	0.6424	0.4029	0.119*	
C27	0.3567 (3)	0.3595 (2)	0.0350 (3)	0.0627 (10)	
H27	0.3237	0.3473	0.0738	0.075*	
C37	0.9318 (3)	0.1585 (2)	0.2232 (2)	0.0605 (9)	
H37	0.8778	0.1601	0.2420	0.073*	
C31	0.5044 (3)	0.4585 (3)	−0.2037 (2)	0.0651 (10)	
H31A	0.5699	0.4965	−0.1936	0.078*	
H31B	0.4547	0.4982	−0.2088	0.078*	
C48	0.1491 (3)	0.3357 (3)	0.3602 (2)	0.0620 (10)	
C28	0.3090 (3)	0.4019 (3)	−0.0402 (3)	0.0691 (11)	
H28	0.2444	0.4167	−0.0506	0.083*	
C29	0.3542 (3)	0.4223 (2)	−0.0994 (3)	0.0629 (10)	
H29	0.3219	0.4508	−0.1491	0.075*	
C45	0.2423 (3)	0.4005 (2)	0.2426 (3)	0.0605 (9)	
C10	0.6735 (5)	0.4346 (4)	0.4583 (4)	0.1015 (16)	
H10A	0.6084	0.4214	0.4058	0.122*	
H10B	0.7041	0.4976	0.4624	0.122*	
C7	0.8883 (4)	0.5317 (3)	0.4038 (3)	0.0927 (15)	
H7	0.8959	0.5440	0.4634	0.111*	
C1	0.6471 (3)	0.2388 (2)	0.2736 (2)	0.0620 (10)	
H1A	0.6787	0.2025	0.3205	0.074*	
H1B	0.5838	0.2515	0.2775	0.074*	
C4	0.8687 (3)	0.4930 (3)	0.2241 (3)	0.0647 (10)	
H4	0.8629	0.4803	0.1651	0.078*	
O6	0.0156 (3)	0.3216 (2)	0.4079 (2)	0.1010 (11)	
N11	0.3791 (3)	0.3097 (3)	0.3130 (4)	0.0902 (12)	
C39	1.0916 (4)	0.1094 (3)	0.2376 (3)	0.0791 (13)	
H39	1.1436	0.0780	0.2682	0.095*	
C50	0.2845 (3)	0.3403 (3)	0.3076 (3)	0.0646 (10)	
N10	0.0986 (4)	0.2994 (3)	0.4162 (2)	0.0800 (11)	
C42	1.0627 (3)	0.2656 (3)	0.0011 (3)	0.0800 (13)	
H42A	1.1329	0.2604	0.0391	0.096*	
H42B	1.0639	0.3281	−0.0109	0.096*	
O9	0.4039 (4)	0.3093 (4)	0.2508 (4)	0.155 (2)	
O5	0.0391 (4)	0.4708 (3)	0.1225 (3)	0.1428 (19)	
C21	0.6869 (4)	−0.1531 (3)	0.1475 (3)	0.0975 (16)	
H21A	0.6654	−0.2035	0.1745	0.117*	
H21B	0.7027	−0.1779	0.0982	0.117*	
C32	0.4687 (5)	0.3971 (4)	−0.2914 (3)	0.1031 (17)	
H32A	0.4723	0.4336	−0.3374	0.124*	
H32B	0.5162	0.3549	−0.2851	0.124*	
C38	1.0110 (3)	0.1127 (3)	0.2646 (3)	0.0760 (12)	
H38	1.0101	0.0827	0.3127	0.091*	
O8	0.4316 (4)	0.2854 (4)	0.3832 (4)	0.1536 (19)	
C22	0.7821 (4)	−0.0933 (4)	0.2167 (4)	0.133 (2)	
H22A	0.8090	−0.0484	0.1882	0.200*	
H22B	0.8333	−0.1295	0.2440	0.200*	
H22C	0.7648	−0.0636	0.2622	0.200*	
O4	0.1450 (4)	0.5715 (3)	0.2179 (3)	0.1296 (15)	
C11	0.6530 (6)	0.4213 (4)	0.5422 (4)	0.130 (2)	
H11A	0.6107	0.3628	0.5334	0.195*	
H11B	0.6177	0.4679	0.5536	0.195*	
H11C	0.7175	0.4249	0.5928	0.195*	
O16	0.8588 (3)	0.1721 (5)	0.7081 (3)	0.174 (2)	
O10	0.7065 (2)	0.2162 (2)	0.76234 (17)	0.0771 (8)	
C51	0.6515 (3)	0.1857 (2)	0.6830 (2)	0.0574 (9)	
C55	0.6367 (3)	0.1493 (3)	0.5255 (2)	0.0612 (9)	
H55	0.6687	0.1480	0.4851	0.073*	
O15	0.8389 (3)	0.2477 (3)	0.6001 (3)	0.1191 (13)	
O11	0.5237 (2)	0.1976 (2)	0.7776 (2)	0.0816 (8)	
C52	0.5414 (3)	0.1541 (2)	0.6456 (2)	0.0591 (9)	
C56	0.6933 (3)	0.1775 (3)	0.6148 (2)	0.0584 (9)	
N12	0.4861 (3)	0.1511 (3)	0.7042 (2)	0.0766 (10)	
N13	0.4700 (3)	0.0922 (3)	0.4015 (2)	0.0812 (11)	
C54	0.5313 (3)	0.1226 (2)	0.4958 (2)	0.0618 (10)	
C53	0.4841 (3)	0.1235 (3)	0.5559 (3)	0.0653 (10)	
H53	0.4131	0.1034	0.5356	0.078*	
O14	0.5141 (3)	0.0912 (3)	0.3502 (2)	0.1057 (12)	
N14	0.8046 (3)	0.2030 (3)	0.6435 (3)	0.0885 (12)	
O12	0.4024 (3)	0.1012 (3)	0.6781 (3)	0.1429 (19)	
O13	0.3766 (3)	0.0677 (3)	0.3776 (2)	0.1141 (13)	
C59	0.8829 (4)	0.1286 (4)	0.4491 (3)	0.0858 (13)	
H59	0.9329	0.1795	0.4578	0.103*	
O17	0.8046 (3)	0.1163 (3)	0.3811 (2)	0.1279 (15)	
N15	0.9016 (3)	0.0747 (3)	0.5112 (2)	0.0774 (10)	
C58	0.9977 (4)	0.0980 (4)	0.5908 (3)	0.1079 (18)	
H58A	1.0419	0.1477	0.5822	0.162*	
H58B	1.0316	0.0463	0.6004	0.162*	
H58C	0.9831	0.1153	0.6423	0.162*	
C57	0.8279 (4)	−0.0031 (4)	0.5063 (4)	0.1088 (17)	
H57A	0.7687	−0.0103	0.4501	0.163*	
H57B	0.8065	0.0050	0.5554	0.163*	
H57C	0.8590	−0.0564	0.5099	0.163*	
C33	0.3641 (5)	0.3450 (5)	−0.3223 (4)	0.146 (3)	
H33A	0.3613	0.3037	−0.2804	0.219*	
H33B	0.3456	0.3113	−0.3807	0.219*	
H33C	0.3166	0.3857	−0.3261	0.219*	
C44	1.0208 (8)	0.1080 (5)	−0.0776 (6)	0.202 (4)	
H44A	1.0793	0.0964	−0.0277	0.303*	
H44B	1.0170	0.0755	−0.1325	0.303*	
H44C	0.9586	0.0880	−0.0687	0.303*	
C43	1.0326 (6)	0.2079 (5)	−0.0837 (4)	0.151 (3)	
H43A	0.9673	0.2203	−0.1256	0.181*	
H43B	1.0840	0.2240	−0.1084	0.181*	

### Atomic displacement parameters (Å^2^)

**Table:**

	*U*^11^	*U*^22^	*U*^33^	*U*^12^	*U*^13^	*U*^23^
Cd1	0.04318 (14)	0.03691 (13)	0.03939 (13)	0.00771 (9)	0.01700 (10)	0.00926 (9)
O1	0.0901 (18)	0.0450 (13)	0.0430 (12)	0.0161 (12)	0.0340 (12)	0.0089 (10)
O2	0.0504 (13)	0.0819 (17)	0.0476 (13)	0.0251 (12)	0.0233 (11)	0.0269 (12)
N5	0.0439 (14)	0.0331 (13)	0.0463 (14)	0.0078 (11)	0.0171 (12)	0.0102 (11)
N4	0.0458 (15)	0.0341 (13)	0.0470 (14)	0.0050 (11)	0.0155 (12)	0.0103 (11)
N3	0.0456 (14)	0.0345 (13)	0.0401 (13)	0.0062 (11)	0.0171 (11)	0.0089 (10)
N1	0.0519 (16)	0.0475 (15)	0.0414 (14)	0.0143 (13)	0.0149 (12)	0.0048 (12)
N7	0.0402 (14)	0.0464 (15)	0.0449 (14)	0.0081 (12)	0.0155 (12)	0.0095 (11)
N6	0.0484 (16)	0.0422 (15)	0.0503 (15)	0.0104 (12)	0.0144 (13)	0.0167 (12)
N8	0.0436 (15)	0.0629 (18)	0.0515 (16)	0.0108 (13)	0.0207 (13)	0.0043 (13)
C24	0.0446 (17)	0.0333 (15)	0.0436 (16)	0.0077 (13)	0.0110 (14)	0.0084 (13)
C13	0.0324 (15)	0.0386 (16)	0.0393 (15)	0.0052 (12)	0.0080 (12)	0.0092 (12)
C14	0.0414 (17)	0.0402 (17)	0.0380 (15)	0.0038 (13)	0.0112 (13)	0.0020 (12)
C23	0.0500 (19)	0.0512 (19)	0.0437 (17)	0.0140 (15)	0.0172 (15)	0.0191 (14)
C19	0.0424 (17)	0.0415 (17)	0.0425 (16)	0.0069 (14)	0.0112 (14)	0.0055 (13)
C25	0.0411 (17)	0.0287 (15)	0.0540 (18)	0.0032 (13)	0.0149 (14)	0.0011 (13)
C35	0.0383 (17)	0.0479 (18)	0.0427 (16)	0.0027 (14)	0.0147 (14)	0.0022 (14)
C12	0.0488 (18)	0.0422 (17)	0.0445 (17)	0.0049 (14)	0.0183 (14)	0.0102 (13)
C40	0.057 (2)	0.074 (3)	0.070 (2)	0.026 (2)	0.0135 (19)	0.000 (2)
C30	0.0444 (18)	0.0368 (17)	0.0585 (19)	0.0071 (14)	0.0149 (15)	0.0082 (14)
C34	0.0473 (19)	0.065 (2)	0.0464 (18)	0.0106 (16)	0.0214 (15)	0.0152 (15)
C36	0.0429 (18)	0.0424 (17)	0.0464 (17)	0.0027 (14)	0.0080 (14)	0.0005 (14)
C9	0.140 (4)	0.077 (3)	0.049 (2)	0.028 (3)	0.032 (3)	0.003 (2)
C18	0.058 (2)	0.0406 (18)	0.062 (2)	0.0090 (16)	0.0184 (17)	0.0016 (15)
C3	0.0439 (19)	0.049 (2)	0.060 (2)	0.0174 (16)	0.0089 (16)	0.0037 (16)
C15	0.067 (2)	0.052 (2)	0.0445 (18)	0.0086 (17)	0.0221 (17)	0.0090 (15)
C41	0.0464 (19)	0.052 (2)	0.0484 (18)	0.0109 (15)	0.0095 (15)	−0.0014 (15)
C26	0.056 (2)	0.0398 (18)	0.062 (2)	0.0074 (15)	0.0249 (17)	0.0068 (15)
N2	0.090 (2)	0.061 (2)	0.0423 (16)	0.0214 (18)	0.0193 (16)	−0.0002 (14)
C2	0.067 (2)	0.051 (2)	0.0390 (17)	0.0260 (17)	0.0160 (16)	0.0048 (14)
O7	0.154 (4)	0.109 (3)	0.073 (2)	−0.001 (2)	0.045 (2)	0.035 (2)
C20	0.075 (3)	0.0435 (19)	0.067 (2)	0.0055 (18)	0.029 (2)	0.0182 (17)
O3	0.106 (2)	0.081 (2)	0.115 (2)	0.0236 (18)	0.079 (2)	0.0353 (18)
C5	0.057 (3)	0.065 (3)	0.127 (4)	0.003 (2)	0.032 (3)	0.003 (3)
C46	0.055 (2)	0.052 (2)	0.062 (2)	0.0019 (16)	0.0240 (18)	0.0107 (16)
C16	0.069 (2)	0.071 (3)	0.0467 (19)	0.0126 (19)	0.0264 (18)	0.0042 (17)
C47	0.059 (2)	0.055 (2)	0.066 (2)	−0.0011 (17)	0.0298 (19)	0.0045 (18)
N9	0.074 (2)	0.071 (2)	0.080 (2)	−0.0010 (19)	0.036 (2)	0.0223 (19)
C49	0.082 (3)	0.047 (2)	0.057 (2)	0.0055 (19)	0.017 (2)	0.0100 (17)
C17	0.065 (2)	0.067 (2)	0.052 (2)	0.0187 (19)	0.0196 (18)	−0.0076 (18)
C8	0.067 (2)	0.057 (2)	0.055 (2)	0.0186 (19)	0.0077 (18)	−0.0056 (17)
C6	0.077 (3)	0.074 (3)	0.114 (4)	0.000 (3)	0.014 (3)	−0.024 (3)
C27	0.061 (2)	0.053 (2)	0.083 (3)	0.0084 (18)	0.039 (2)	0.0047 (19)
C37	0.061 (2)	0.058 (2)	0.055 (2)	0.0074 (18)	0.0139 (17)	0.0173 (17)
C31	0.066 (2)	0.065 (2)	0.062 (2)	0.0199 (19)	0.0173 (19)	0.0303 (19)
C48	0.078 (3)	0.053 (2)	0.053 (2)	−0.0021 (19)	0.0301 (19)	0.0024 (17)
C28	0.052 (2)	0.062 (2)	0.099 (3)	0.0213 (19)	0.032 (2)	0.013 (2)
C29	0.052 (2)	0.056 (2)	0.080 (3)	0.0195 (18)	0.0204 (19)	0.0203 (19)
C45	0.067 (2)	0.049 (2)	0.068 (2)	0.0018 (18)	0.033 (2)	0.0064 (17)
C10	0.136 (5)	0.095 (4)	0.091 (3)	0.024 (3)	0.065 (3)	0.002 (3)
C7	0.089 (3)	0.078 (3)	0.081 (3)	0.011 (3)	0.008 (3)	−0.023 (3)
C1	0.093 (3)	0.056 (2)	0.050 (2)	0.021 (2)	0.039 (2)	0.0073 (16)
C4	0.049 (2)	0.058 (2)	0.084 (3)	0.0136 (18)	0.022 (2)	0.005 (2)
O6	0.131 (3)	0.093 (2)	0.108 (3)	0.002 (2)	0.086 (3)	0.0066 (19)
N11	0.089 (3)	0.069 (2)	0.118 (4)	0.026 (2)	0.042 (3)	0.024 (2)
C39	0.071 (3)	0.070 (3)	0.078 (3)	0.030 (2)	0.003 (2)	0.014 (2)
C50	0.064 (2)	0.049 (2)	0.081 (3)	0.0105 (18)	0.029 (2)	0.0046 (19)
N10	0.112 (3)	0.065 (2)	0.064 (2)	−0.007 (2)	0.044 (2)	0.0070 (18)
C42	0.063 (3)	0.114 (4)	0.080 (3)	0.026 (2)	0.044 (2)	0.014 (3)
O9	0.151 (4)	0.203 (5)	0.188 (5)	0.109 (4)	0.114 (4)	0.081 (4)
O5	0.151 (4)	0.106 (3)	0.096 (3)	−0.003 (3)	−0.025 (3)	0.026 (2)
C21	0.113 (4)	0.083 (3)	0.089 (3)	0.027 (3)	0.024 (3)	0.036 (3)
C32	0.129 (5)	0.094 (4)	0.066 (3)	0.032 (3)	0.011 (3)	0.028 (3)
C38	0.075 (3)	0.069 (3)	0.067 (2)	0.015 (2)	0.006 (2)	0.023 (2)
O8	0.122 (4)	0.160 (4)	0.189 (5)	0.077 (3)	0.047 (3)	0.075 (4)
C22	0.119 (5)	0.124 (5)	0.117 (5)	0.035 (4)	−0.001 (4)	0.017 (4)
O4	0.163 (4)	0.074 (2)	0.126 (3)	0.015 (3)	0.029 (3)	0.034 (2)
C11	0.178 (6)	0.129 (5)	0.093 (4)	0.006 (5)	0.077 (4)	−0.016 (4)
O16	0.091 (3)	0.351 (8)	0.093 (3)	0.072 (4)	0.030 (2)	0.078 (4)
O10	0.0792 (19)	0.093 (2)	0.0483 (15)	−0.0069 (16)	0.0229 (14)	−0.0034 (14)
C51	0.073 (2)	0.048 (2)	0.050 (2)	0.0057 (18)	0.0248 (18)	0.0122 (16)
C55	0.074 (3)	0.065 (2)	0.048 (2)	0.011 (2)	0.0276 (19)	0.0118 (17)
O15	0.084 (2)	0.146 (4)	0.134 (3)	0.000 (2)	0.055 (2)	0.036 (3)
O11	0.091 (2)	0.091 (2)	0.0687 (18)	0.0056 (17)	0.0435 (17)	0.0024 (16)
C52	0.072 (2)	0.056 (2)	0.052 (2)	0.0022 (18)	0.0310 (18)	0.0086 (16)
C56	0.060 (2)	0.065 (2)	0.052 (2)	0.0084 (18)	0.0239 (17)	0.0137 (17)
N12	0.077 (2)	0.081 (2)	0.068 (2)	−0.007 (2)	0.0335 (19)	0.0014 (19)
N13	0.092 (3)	0.082 (3)	0.050 (2)	−0.003 (2)	0.015 (2)	0.0058 (17)
C54	0.077 (3)	0.054 (2)	0.0451 (19)	0.0025 (19)	0.0174 (18)	0.0067 (16)
C53	0.070 (3)	0.057 (2)	0.061 (2)	−0.0025 (19)	0.022 (2)	0.0076 (18)
O14	0.118 (3)	0.139 (3)	0.0493 (17)	0.002 (2)	0.0315 (18)	−0.0001 (18)
N14	0.075 (3)	0.133 (4)	0.058 (2)	0.019 (2)	0.028 (2)	0.014 (2)
O12	0.110 (3)	0.194 (4)	0.108 (3)	−0.066 (3)	0.068 (2)	−0.041 (3)
O13	0.091 (3)	0.147 (3)	0.067 (2)	−0.023 (2)	0.0081 (18)	−0.005 (2)
C59	0.080 (3)	0.095 (3)	0.072 (3)	0.011 (3)	0.019 (3)	0.021 (3)
O17	0.104 (3)	0.151 (4)	0.084 (2)	0.012 (3)	−0.012 (2)	0.039 (2)
N15	0.072 (2)	0.085 (3)	0.064 (2)	0.0166 (19)	0.0122 (17)	0.0171 (18)
C58	0.092 (4)	0.133 (5)	0.078 (3)	0.038 (3)	0.004 (3)	0.018 (3)
C57	0.105 (4)	0.101 (4)	0.115 (4)	0.004 (3)	0.041 (3)	0.026 (3)
C33	0.129 (6)	0.127 (5)	0.119 (5)	0.007 (4)	−0.013 (4)	0.018 (4)
C44	0.251 (11)	0.190 (7)	0.152 (7)	−0.007 (9)	0.096 (7)	−0.059 (7)
C43	0.139 (6)	0.214 (7)	0.111 (5)	0.028 (6)	0.072 (5)	−0.026 (6)

### Geometric parameters (Å, °)

**Table:**

Cd1—N5	2.233 (2)	C8—C7	1.384 (6)
Cd1—N1	2.242 (3)	C6—C7	1.366 (7)
Cd1—N3	2.250 (3)	C6—H6	0.9300
Cd1—N7	2.263 (3)	C27—C28	1.396 (6)
Cd1—O2	2.561 (2)	C27—H27	0.9300
Cd1—O1	2.618 (2)	C37—C38	1.377 (5)
O1—C1	1.414 (4)	C37—H37	0.9300
O1—C12	1.416 (4)	C31—C32	1.500 (6)
O2—C23	1.416 (4)	C31—H31A	0.9700
O2—C34	1.412 (4)	C31—H31B	0.9700
N5—C24	1.321 (4)	C48—N10	1.447 (5)
N5—C25	1.391 (4)	C28—C29	1.372 (6)
N4—C13	1.353 (4)	C28—H28	0.9300
N4—C19	1.386 (4)	C29—H29	0.9300
N4—C20	1.462 (4)	C45—C50	1.445 (5)
N3—C13	1.314 (4)	C10—C11	1.516 (7)
N3—C14	1.394 (4)	C10—H10A	0.9700
N1—C2	1.319 (4)	C10—H10B	0.9700
N1—C3	1.390 (4)	C7—H7	0.9300
N7—C35	1.316 (4)	C1—H1A	0.9700
N7—C36	1.388 (4)	C1—H1B	0.9700
N6—C24	1.353 (4)	C4—H4	0.9300
N6—C30	1.382 (4)	O6—N10	1.230 (5)
N6—C31	1.481 (4)	N11—O9	1.188 (6)
N8—C35	1.354 (4)	N11—O8	1.213 (6)
N8—C41	1.383 (4)	N11—C50	1.454 (6)
N8—C42	1.464 (5)	C39—C38	1.379 (6)
C24—C23	1.485 (4)	C39—H39	0.9300
C13—C12	1.488 (4)	C42—C43	1.445 (7)
C14—C15	1.388 (4)	C42—H42A	0.9700
C14—C19	1.385 (4)	C42—H42B	0.9700
C23—H23A	0.9700	C21—C22	1.4995 (7)
C23—H23B	0.9700	C21—H21A	0.9700
C19—C18	1.390 (4)	C21—H21B	0.9700
C25—C26	1.388 (5)	C32—C33	1.456 (8)
C25—C30	1.392 (4)	C32—H32A	0.9700
C35—C34	1.490 (4)	C32—H32B	0.9700
C12—H12A	0.9700	C38—H38	0.9300
C12—H12B	0.9700	C22—H22A	0.9600
C40—C39	1.372 (6)	C22—H22B	0.9600
C40—C41	1.395 (5)	C22—H22C	0.9600
C40—H40	0.9300	C11—H11A	0.9600
C30—C29	1.385 (5)	C11—H11B	0.9600
C34—H34A	0.9700	C11—H11C	0.9600
C34—H34B	0.9700	O16—N14	1.206 (5)
C36—C41	1.387 (5)	O10—C51	1.232 (4)
C36—C37	1.392 (5)	C51—C52	1.438 (5)
C9—N2	1.483 (5)	C51—C56	1.445 (5)
C9—C10	1.507 (7)	C55—C56	1.357 (5)
C9—H9A	0.9700	C55—C54	1.374 (5)
C9—H9B	0.9700	C55—H55	0.9300
C18—C17	1.364 (5)	O15—N14	1.192 (5)
C18—H18	0.9300	O11—N12	1.215 (4)
C3—C4	1.373 (5)	C52—C53	1.369 (5)
C3—C8	1.393 (5)	C52—N12	1.445 (5)
C15—C16	1.368 (5)	C56—N14	1.452 (5)
C15—H15	0.9300	N12—O12	1.215 (5)
C26—C27	1.371 (5)	N13—O14	1.217 (5)
C26—H26	0.9300	N13—O13	1.224 (5)
N2—C2	1.345 (4)	N13—C54	1.438 (5)
N2—C8	1.377 (5)	C54—C53	1.377 (5)
C2—C1	1.479 (5)	C53—H53	0.9300
O7—N10	1.216 (5)	C59—O17	1.209 (5)
C20—C21	1.461 (6)	C59—N15	1.307 (5)
C20—H20A	0.9700	C59—H59	0.9300
C20—H20B	0.9700	N15—C57	1.432 (6)
O3—C45	1.235 (4)	N15—C58	1.451 (5)
C5—C6	1.386 (7)	C58—H58A	0.9600
C5—C4	1.382 (6)	C58—H58B	0.9600
C5—H5	0.9300	C58—H58C	0.9600
C46—C47	1.346 (5)	C57—H57A	0.9600
C46—C45	1.440 (5)	C57—H57B	0.9600
C46—N9	1.471 (5)	C57—H57C	0.9600
C16—C17	1.396 (5)	C33—H33A	0.9600
C16—H16	0.9300	C33—H33B	0.9600
C47—C48	1.376 (5)	C33—H33C	0.9600
C47—H47	0.9300	C44—C43	1.4997 (9)
N9—O5	1.133 (5)	C44—H44A	0.9600
N9—O4	1.204 (5)	C44—H44B	0.9600
C49—C48	1.374 (6)	C44—H44C	0.9600
C49—C50	1.374 (6)	C43—H43A	0.9700
C49—H49	0.9300	C43—H43B	0.9700
C17—H17	0.9300		
			
N5—Cd1—N1	102.14 (9)	C28—C27—H27	119.6
N5—Cd1—N3	108.82 (9)	C38—C37—C36	116.4 (4)
N1—Cd1—N3	127.24 (9)	C38—C37—H37	121.8
N5—Cd1—N7	131.02 (9)	C36—C37—H37	121.8
N1—Cd1—N7	101.21 (9)	N6—C31—C32	114.9 (3)
N3—Cd1—N7	89.53 (9)	N6—C31—H31A	108.5
N5—Cd1—O2	65.77 (8)	C32—C31—H31A	108.5
N1—Cd1—O2	115.93 (10)	N6—C31—H31B	108.5
N3—Cd1—O2	115.47 (9)	C32—C31—H31B	108.5
N7—Cd1—O2	65.34 (8)	H31A—C31—H31B	107.5
N5—Cd1—O1	107.15 (9)	C49—C48—C47	120.8 (4)
N1—Cd1—O1	65.86 (9)	C49—C48—N10	119.9 (4)
N3—Cd1—O1	64.71 (8)	C47—C48—N10	119.3 (4)
N7—Cd1—O1	121.64 (8)	C29—C28—C27	122.2 (4)
O2—Cd1—O1	172.80 (7)	C29—C28—H28	118.9
C1—O1—C12	115.2 (2)	C27—C28—H28	118.9
C1—O1—Cd1	117.3 (2)	C28—C29—C30	116.9 (3)
C12—O1—Cd1	116.95 (17)	C28—C29—H29	121.6
C23—O2—C34	115.5 (2)	C30—C29—H29	121.6
C23—O2—Cd1	117.99 (18)	O3—C45—C46	121.8 (4)
C34—O2—Cd1	119.18 (18)	O3—C45—C50	127.5 (4)
C24—N5—C25	105.9 (2)	C46—C45—C50	110.7 (3)
C24—N5—Cd1	124.2 (2)	C9—C10—C11	111.9 (5)
C25—N5—Cd1	129.8 (2)	C9—C10—H10A	109.2
C13—N4—C19	107.2 (2)	C11—C10—H10A	109.2
C13—N4—C20	126.9 (3)	C9—C10—H10B	109.2
C19—N4—C20	125.8 (3)	C11—C10—H10B	109.2
C13—N3—C14	105.9 (2)	H10A—C10—H10B	107.9
C13—N3—Cd1	124.8 (2)	C6—C7—C8	117.0 (5)
C14—N3—Cd1	127.45 (19)	C6—C7—H7	121.5
C2—N1—C3	106.2 (3)	C8—C7—H7	121.5
C2—N1—Cd1	124.9 (2)	O1—C1—C2	107.2 (3)
C3—N1—Cd1	128.6 (2)	O1—C1—H1A	110.3
C35—N7—C36	105.6 (3)	C2—C1—H1A	110.3
C35—N7—Cd1	124.2 (2)	O1—C1—H1B	110.3
C36—N7—Cd1	129.3 (2)	C2—C1—H1B	110.3
C24—N6—C30	106.8 (3)	H1A—C1—H1B	108.5
C24—N6—C31	126.3 (3)	C3—C4—C5	117.4 (4)
C30—N6—C31	126.8 (3)	C3—C4—H4	121.3
C35—N8—C41	106.8 (3)	C5—C4—H4	121.3
C35—N8—C42	126.4 (3)	O9—N11—O8	121.5 (5)
C41—N8—C42	126.8 (3)	O9—N11—C50	120.8 (5)
N5—C24—N6	112.4 (3)	O8—N11—C50	117.7 (5)
N5—C24—C23	123.7 (3)	C40—C39—C38	121.6 (4)
N6—C24—C23	123.8 (3)	C40—C39—H39	119.2
N3—C13—N4	112.2 (3)	C38—C39—H39	119.2
N3—C13—C12	123.8 (3)	C49—C50—N11	118.0 (4)
N4—C13—C12	124.1 (3)	C49—C50—C45	123.6 (4)
C15—C14—C19	120.8 (3)	N11—C50—C45	118.5 (4)
C15—C14—N3	130.2 (3)	O7—N10—O6	123.8 (4)
C19—C14—N3	109.0 (3)	O7—N10—C48	118.4 (5)
O2—C23—C24	105.4 (2)	O6—N10—C48	117.8 (4)
O2—C23—H23A	110.7	N8—C42—C43	117.0 (4)
C24—C23—H23A	110.7	N8—C42—H42A	108.0
O2—C23—H23B	110.7	C43—C42—H42A	108.0
C24—C23—H23B	110.7	N8—C42—H42B	108.0
H23A—C23—H23B	108.8	C43—C42—H42B	108.0
N4—C19—C14	105.7 (3)	H42A—C42—H42B	107.3
N4—C19—C18	132.5 (3)	C22—C21—C20	111.3 (4)
C14—C19—C18	121.7 (3)	C22—C21—H21A	109.4
C26—C25—N5	130.4 (3)	C20—C21—H21A	109.4
C26—C25—C30	121.1 (3)	C22—C21—H21B	109.4
N5—C25—C30	108.5 (3)	C20—C21—H21B	109.4
N7—C35—N8	112.6 (3)	H21A—C21—H21B	108.0
N7—C35—C34	123.5 (3)	C33—C32—C31	114.9 (6)
N8—C35—C34	123.9 (3)	C33—C32—H32A	108.5
O1—C12—C13	106.0 (2)	C31—C32—H32A	108.5
O1—C12—H12A	110.5	C33—C32—H32B	108.5
C13—C12—H12A	110.5	C31—C32—H32B	108.5
O1—C12—H12B	110.5	H32A—C32—H32B	107.5
C13—C12—H12B	110.5	C37—C38—C39	122.6 (4)
H12A—C12—H12B	108.7	C37—C38—H38	118.7
C39—C40—C41	116.6 (4)	C39—C38—H38	118.7
C39—C40—H40	121.7	C21—C22—H22A	109.5
C41—C40—H40	121.7	C21—C22—H22B	109.5
N6—C30—C29	132.3 (3)	H22A—C22—H22B	109.5
N6—C30—C25	106.3 (3)	C21—C22—H22C	109.5
C29—C30—C25	121.3 (3)	H22A—C22—H22C	109.5
O2—C34—C35	105.8 (2)	H22B—C22—H22C	109.5
O2—C34—H34A	110.6	C10—C11—H11A	109.5
C35—C34—H34A	110.6	C10—C11—H11B	109.5
O2—C34—H34B	110.6	H11A—C11—H11B	109.5
C35—C34—H34B	110.6	C10—C11—H11C	109.5
H34A—C34—H34B	108.7	H11A—C11—H11C	109.5
N7—C36—C41	109.1 (3)	H11B—C11—H11C	109.5
N7—C36—C37	129.8 (3)	O10—C51—C52	126.5 (3)
C41—C36—C37	121.1 (3)	O10—C51—C56	122.2 (4)
N2—C9—C10	112.7 (4)	C52—C51—C56	111.3 (3)
N2—C9—H9A	109.1	C56—C55—C54	119.0 (4)
C10—C9—H9A	109.1	C56—C55—H55	120.5
N2—C9—H9B	109.1	C54—C55—H55	120.5
C10—C9—H9B	109.1	C53—C52—C51	123.6 (3)
H9A—C9—H9B	107.8	C53—C52—N12	116.6 (4)
C17—C18—C19	116.7 (3)	C51—C52—N12	119.8 (3)
C17—C18—H18	121.6	C55—C56—C51	125.2 (4)
C19—C18—H18	121.6	C55—C56—N14	117.2 (3)
C4—C3—N1	130.5 (3)	C51—C56—N14	117.5 (3)
C4—C3—C8	121.3 (4)	O12—N12—O11	121.7 (4)
N1—C3—C8	108.1 (3)	O12—N12—C52	118.4 (4)
C16—C15—C14	117.7 (3)	O11—N12—C52	119.9 (3)
C16—C15—H15	121.2	O14—N13—O13	123.7 (4)
C14—C15—H15	121.2	O14—N13—C54	118.1 (4)
N8—C41—C36	105.9 (3)	O13—N13—C54	118.1 (4)
N8—C41—C40	132.4 (4)	C55—C54—C53	120.4 (3)
C36—C41—C40	121.7 (4)	C55—C54—N13	119.9 (4)
C27—C26—C25	117.6 (3)	C53—C54—N13	119.7 (4)
C27—C26—H26	121.2	C52—C53—C54	120.2 (4)
C25—C26—H26	121.2	C52—C53—H53	119.9
C2—N2—C8	107.2 (3)	C54—C53—H53	119.9
C2—N2—C9	126.9 (4)	O15—N14—O16	122.6 (5)
C8—N2—C9	125.9 (3)	O15—N14—C56	119.8 (4)
N1—C2—N2	112.2 (3)	O16—N14—C56	117.4 (4)
N1—C2—C1	124.6 (3)	O17—C59—N15	124.0 (5)
N2—C2—C1	123.2 (3)	O17—C59—H59	118.0
N4—C20—C21	115.2 (3)	N15—C59—H59	118.0
N4—C20—H20A	108.5	C59—N15—C57	122.4 (4)
C21—C20—H20A	108.5	C59—N15—C58	118.7 (4)
N4—C20—H20B	108.5	C57—N15—C58	118.7 (4)
C21—C20—H20B	108.5	N15—C58—H58A	109.5
H20A—C20—H20B	107.5	N15—C58—H58B	109.5
C6—C5—C4	121.0 (5)	H58A—C58—H58B	109.5
C6—C5—H5	119.5	N15—C58—H58C	109.5
C4—C5—H5	119.5	H58A—C58—H58C	109.5
C47—C46—C45	126.4 (4)	H58B—C58—H58C	109.5
C47—C46—N9	118.3 (3)	N15—C57—H57A	109.5
C45—C46—N9	115.3 (3)	N15—C57—H57B	109.5
C15—C16—C17	121.0 (3)	H57A—C57—H57B	109.5
C15—C16—H16	119.5	N15—C57—H57C	109.5
C17—C16—H16	119.5	H57A—C57—H57C	109.5
C46—C47—C48	118.5 (4)	H57B—C57—H57C	109.5
C46—C47—H47	120.8	C32—C33—H33A	109.5
C48—C47—H47	120.8	C32—C33—H33B	109.5
O5—N9—O4	122.0 (4)	H33A—C33—H33B	109.5
O5—N9—C46	121.0 (4)	C32—C33—H33C	109.5
O4—N9—C46	117.0 (4)	H33A—C33—H33C	109.5
C48—C49—C50	119.9 (4)	H33B—C33—H33C	109.5
C48—C49—H49	120.1	C43—C44—H44A	109.5
C50—C49—H49	120.1	C43—C44—H44B	109.5
C18—C17—C16	122.1 (3)	H44A—C44—H44B	109.5
C18—C17—H17	119.0	C43—C44—H44C	109.5
C16—C17—H17	119.0	H44A—C44—H44C	109.5
N2—C8—C7	132.5 (4)	H44B—C44—H44C	109.5
N2—C8—C3	106.3 (3)	C44—C43—C42	114.7 (6)
C7—C8—C3	121.2 (5)	C44—C43—H43A	108.6
C7—C6—C5	122.1 (5)	C42—C43—H43A	108.6
C7—C6—H6	118.9	C44—C43—H43B	108.6
C5—C6—H6	118.9	C42—C43—H43B	108.6
C26—C27—C28	120.9 (4)	H43A—C43—H43B	107.6
C26—C27—H27	119.6		
			
N5—Cd1—O1—C1	−96.2 (2)	Cd1—N1—C3—C4	7.0 (5)
N1—Cd1—O1—C1	−0.3 (2)	C2—N1—C3—C8	0.2 (4)
N3—Cd1—O1—C1	160.6 (3)	Cd1—N1—C3—C8	−173.5 (2)
N7—Cd1—O1—C1	88.2 (2)	C19—C14—C15—C16	0.2 (5)
O2—Cd1—O1—C1	−106.3 (6)	N3—C14—C15—C16	−178.1 (3)
N5—Cd1—O1—C12	120.6 (2)	C35—N8—C41—C36	−0.5 (3)
N1—Cd1—O1—C12	−143.5 (2)	C42—N8—C41—C36	178.7 (3)
N3—Cd1—O1—C12	17.4 (2)	C35—N8—C41—C40	178.9 (4)
N7—Cd1—O1—C12	−55.0 (2)	C42—N8—C41—C40	−1.8 (6)
O2—Cd1—O1—C12	110.5 (6)	N7—C36—C41—N8	−0.1 (4)
N5—Cd1—O2—C23	−15.5 (2)	C37—C36—C41—N8	179.6 (3)
N1—Cd1—O2—C23	−107.6 (2)	N7—C36—C41—C40	−179.7 (3)
N3—Cd1—O2—C23	84.7 (2)	C37—C36—C41—C40	0.0 (5)
N7—Cd1—O2—C23	161.5 (2)	C39—C40—C41—N8	180.0 (4)
O1—Cd1—O2—C23	−4.9 (7)	C39—C40—C41—C36	−0.6 (5)
N5—Cd1—O2—C34	−164.2 (3)	N5—C25—C26—C27	178.6 (3)
N1—Cd1—O2—C34	103.7 (2)	C30—C25—C26—C27	0.7 (5)
N3—Cd1—O2—C34	−64.0 (2)	C10—C9—N2—C2	−103.1 (5)
N7—Cd1—O2—C34	12.8 (2)	C10—C9—N2—C8	75.9 (6)
O1—Cd1—O2—C34	−153.7 (5)	C3—N1—C2—N2	−0.3 (4)
N1—Cd1—N5—C24	123.1 (2)	Cd1—N1—C2—N2	173.7 (2)
N3—Cd1—N5—C24	−100.2 (2)	C3—N1—C2—C1	−179.6 (3)
N7—Cd1—N5—C24	6.3 (3)	Cd1—N1—C2—C1	−5.6 (5)
O2—Cd1—N5—C24	9.9 (2)	C8—N2—C2—N1	0.3 (4)
O1—Cd1—N5—C24	−168.7 (2)	C9—N2—C2—N1	179.5 (4)
N1—Cd1—N5—C25	−59.3 (3)	C8—N2—C2—C1	179.6 (3)
N3—Cd1—N5—C25	77.3 (3)	C9—N2—C2—C1	−1.2 (6)
N7—Cd1—N5—C25	−176.1 (2)	C13—N4—C20—C21	−113.5 (4)
O2—Cd1—N5—C25	−172.5 (3)	C19—N4—C20—C21	63.6 (5)
O1—Cd1—N5—C25	8.9 (3)	C14—C15—C16—C17	0.6 (5)
N5—Cd1—N3—C13	−115.9 (2)	C45—C46—C47—C48	2.6 (6)
N1—Cd1—N3—C13	6.7 (3)	N9—C46—C47—C48	−176.2 (3)
N7—Cd1—N3—C13	110.5 (2)	C47—C46—N9—O5	−83.6 (6)
O2—Cd1—N3—C13	172.7 (2)	C45—C46—N9—O5	97.4 (5)
O1—Cd1—N3—C13	−15.3 (2)	C47—C46—N9—O4	97.4 (5)
N5—Cd1—N3—C14	81.8 (2)	C45—C46—N9—O4	−81.6 (5)
N1—Cd1—N3—C14	−155.6 (2)	C19—C18—C17—C16	0.9 (5)
N7—Cd1—N3—C14	−51.8 (2)	C15—C16—C17—C18	−1.2 (6)
O2—Cd1—N3—C14	10.4 (3)	C2—N2—C8—C7	−178.2 (4)
O1—Cd1—N3—C14	−177.6 (3)	C9—N2—C8—C7	2.6 (7)
N5—Cd1—N1—C2	106.5 (3)	C2—N2—C8—C3	−0.2 (4)
N3—Cd1—N1—C2	−18.8 (3)	C9—N2—C8—C3	−179.4 (4)
N7—Cd1—N1—C2	−116.9 (3)	C4—C3—C8—N2	179.5 (3)
O2—Cd1—N1—C2	175.2 (2)	N1—C3—C8—N2	0.0 (4)
O1—Cd1—N1—C2	2.9 (2)	C4—C3—C8—C7	−2.2 (6)
N5—Cd1—N1—C3	−80.9 (3)	N1—C3—C8—C7	178.3 (4)
N3—Cd1—N1—C3	153.8 (2)	C4—C5—C6—C7	−0.7 (8)
N7—Cd1—N1—C3	55.7 (3)	C25—C26—C27—C28	0.0 (5)
O2—Cd1—N1—C3	−12.1 (3)	N7—C36—C37—C38	179.9 (3)
O1—Cd1—N1—C3	175.6 (3)	C41—C36—C37—C38	0.3 (5)
N5—Cd1—N7—C35	−6.6 (3)	C24—N6—C31—C32	82.8 (5)
N1—Cd1—N7—C35	−123.8 (2)	C30—N6—C31—C32	−101.1 (5)
N3—Cd1—N7—C35	108.3 (3)	C50—C49—C48—C47	−1.8 (6)
O2—Cd1—N7—C35	−10.2 (2)	C50—C49—C48—N10	176.6 (3)
O1—Cd1—N7—C35	167.8 (2)	C46—C47—C48—C49	0.5 (6)
N5—Cd1—N7—C36	−174.0 (2)	C46—C47—C48—N10	−178.0 (3)
N1—Cd1—N7—C36	68.9 (3)	C26—C27—C28—C29	−0.5 (6)
N3—Cd1—N7—C36	−59.1 (3)	C27—C28—C29—C30	0.3 (6)
O2—Cd1—N7—C36	−177.6 (3)	N6—C30—C29—C28	−177.6 (4)
O1—Cd1—N7—C36	0.4 (3)	C25—C30—C29—C28	0.4 (5)
C25—N5—C24—N6	−1.1 (3)	C47—C46—C45—O3	176.9 (4)
Cd1—N5—C24—N6	176.98 (19)	N9—C46—C45—O3	−4.2 (5)
C25—N5—C24—C23	177.6 (3)	C47—C46—C45—C50	−3.8 (5)
Cd1—N5—C24—C23	−4.4 (4)	N9—C46—C45—C50	175.1 (3)
C30—N6—C24—N5	0.6 (3)	N2—C9—C10—C11	177.2 (5)
C31—N6—C24—N5	177.3 (3)	C5—C6—C7—C8	−0.9 (8)
C30—N6—C24—C23	−178.0 (3)	N2—C8—C7—C6	−180.0 (5)
C31—N6—C24—C23	−1.3 (5)	C3—C8—C7—C6	2.3 (7)
C14—N3—C13—N4	−0.6 (3)	C12—O1—C1—C2	142.1 (3)
Cd1—N3—C13—N4	−166.06 (18)	Cd1—O1—C1—C2	−1.7 (4)
C14—N3—C13—C12	178.4 (3)	N1—C2—C1—O1	4.5 (5)
Cd1—N3—C13—C12	12.9 (4)	N2—C2—C1—O1	−174.7 (3)
C19—N4—C13—N3	0.6 (3)	N1—C3—C4—C5	179.9 (3)
C20—N4—C13—N3	178.2 (3)	C8—C3—C4—C5	0.6 (5)
C19—N4—C13—C12	−178.4 (3)	C6—C5—C4—C3	0.9 (6)
C20—N4—C13—C12	−0.8 (5)	C41—C40—C39—C38	0.9 (6)
C13—N3—C14—C15	178.9 (3)	C48—C49—C50—N11	−179.0 (4)
Cd1—N3—C14—C15	−16.2 (5)	C48—C49—C50—C45	0.3 (6)
C13—N3—C14—C19	0.4 (3)	O9—N11—C50—C49	156.4 (5)
Cd1—N3—C14—C19	165.3 (2)	O8—N11—C50—C49	−24.1 (6)
C34—O2—C23—C24	167.1 (3)	O9—N11—C50—C45	−22.9 (7)
Cd1—O2—C23—C24	17.3 (3)	O8—N11—C50—C45	156.5 (5)
N5—C24—C23—O2	−9.7 (4)	O3—C45—C50—C49	−178.5 (4)
N6—C24—C23—O2	168.8 (3)	C46—C45—C50—C49	2.3 (5)
C13—N4—C19—C14	−0.3 (3)	O3—C45—C50—N11	0.8 (6)
C20—N4—C19—C14	−177.9 (3)	C46—C45—C50—N11	−178.5 (3)
C13—N4—C19—C18	−178.2 (3)	C49—C48—N10—O7	1.9 (6)
C20—N4—C19—C18	4.2 (6)	C47—C48—N10—O7	−179.6 (4)
C15—C14—C19—N4	−178.7 (3)	C49—C48—N10—O6	−177.0 (4)
N3—C14—C19—N4	0.0 (3)	C47—C48—N10—O6	1.5 (5)
C15—C14—C19—C18	−0.5 (5)	C35—N8—C42—C43	−82.5 (6)
N3—C14—C19—C18	178.2 (3)	C41—N8—C42—C43	98.4 (5)
C24—N5—C25—C26	−177.0 (3)	N4—C20—C21—C22	58.3 (6)
Cd1—N5—C25—C26	5.1 (5)	N6—C31—C32—C33	66.8 (6)
C24—N5—C25—C30	1.1 (3)	C36—C37—C38—C39	0.0 (6)
Cd1—N5—C25—C30	−176.8 (2)	C40—C39—C38—C37	−0.6 (7)
C36—N7—C35—N8	−1.1 (3)	O10—C51—C52—C53	177.8 (4)
Cd1—N7—C35—N8	−171.0 (2)	C56—C51—C52—C53	−2.4 (5)
C36—N7—C35—C34	177.5 (3)	O10—C51—C52—N12	−3.9 (6)
Cd1—N7—C35—C34	7.7 (4)	C56—C51—C52—N12	175.9 (3)
C41—N8—C35—N7	1.1 (4)	C54—C55—C56—C51	−2.6 (6)
C42—N8—C35—N7	−178.2 (3)	C54—C55—C56—N14	178.6 (4)
C41—N8—C35—C34	−177.6 (3)	O10—C51—C56—C55	−176.2 (4)
C42—N8—C35—C34	3.1 (5)	C52—C51—C56—C55	4.0 (5)
C1—O1—C12—C13	−160.5 (3)	O10—C51—C56—N14	2.6 (6)
Cd1—O1—C12—C13	−16.6 (3)	C52—C51—C56—N14	−177.2 (4)
N3—C13—C12—O1	4.6 (4)	C53—C52—N12—O12	19.6 (6)
N4—C13—C12—O1	−176.5 (3)	C51—C52—N12—O12	−158.7 (5)
C24—N6—C30—C29	178.4 (4)	C53—C52—N12—O11	−160.5 (4)
C31—N6—C30—C29	1.7 (6)	C51—C52—N12—O11	21.1 (6)
C24—N6—C30—C25	0.1 (3)	C56—C55—C54—C53	−0.7 (6)
C31—N6—C30—C25	−176.6 (3)	C56—C55—C54—N13	−179.8 (4)
C26—C25—C30—N6	177.6 (3)	O14—N13—C54—C55	−0.1 (6)
N5—C25—C30—N6	−0.7 (3)	O13—N13—C54—C55	179.0 (4)
C26—C25—C30—C29	−0.9 (5)	O14—N13—C54—C53	−179.3 (4)
N5—C25—C30—C29	−179.2 (3)	O13—N13—C54—C53	−0.1 (6)
C23—O2—C34—C35	−162.3 (3)	C51—C52—C53—C54	−0.5 (6)
Cd1—O2—C34—C35	−12.8 (3)	N12—C52—C53—C54	−178.8 (4)
N7—C35—C34—O2	4.5 (4)	C55—C54—C53—C52	2.2 (6)
N8—C35—C34—O2	−177.0 (3)	N13—C54—C53—C52	−178.7 (4)
C35—N7—C36—C41	0.7 (3)	C55—C56—N14—O15	43.0 (6)
Cd1—N7—C36—C41	169.9 (2)	C51—C56—N14—O15	−135.9 (5)
C35—N7—C36—C37	−178.9 (3)	C55—C56—N14—O16	−132.0 (5)
Cd1—N7—C36—C37	−9.7 (5)	C51—C56—N14—O16	49.1 (6)
N4—C19—C18—C17	177.6 (3)	O17—C59—N15—C57	−2.7 (8)
C14—C19—C18—C17	−0.1 (5)	O17—C59—N15—C58	−178.8 (5)
C2—N1—C3—C4	−179.3 (4)	N8—C42—C43—C44	−54.4 (9)
